# Double-blind, randomized pilot study of bioadhesive chlorhexidine gel in the prevention and treatment of mucositis induced by chemoradiotherapy of head and neck cancer

**DOI:** 10.4317/medoral.20338

**Published:** 2015-02-07

**Authors:** Rosa-Maria Diaz-Sanchez, Jerónimo Pachón-Ibáñez, Fátima Marín-Conde, Ángela Rodríguez-Caballero, Jose-Luis Gutierrez-Perez, Daniel Torres-Lagares

**Affiliations:** 1 Dental School. University of Seville; 2 “Virgen del Rocío” University Hospital

## Abstract

**Background:**

To evaluate, in an initial way, the effectiveness of bioadhesive chlorhexidine gel 0.2% versus placebo as a preventive and therapeutic intervention of oral mucositis induced by radiation therapy and chemotherapy in patients diagnosed with head and neck cancer treated with chemoradiotherapy.

**Material and Methods:**

In this pilot study, 7 patients (range of age: 18- 65), having histological documented diagnosis of squamous carcinoma on the head and neck region in stage III and IV, and receiving combined radiation treatment and chemotherapy (cisplatin 100 mg/m2 IV on days 1, 22, and 43 of irradiation) were studied. Simultaneously, a topical application was performed with bioadhesive chlorhexidine gel 0.2% in the study group, and the placebo gel for the control group in 5 applications per day, from the time of initiation of cancer treatment to 2 weeks after completion of chemo-radiotherapy treatment (11 weeks of follow-up). The gradation of mucositis, pain, analgesic consumption, infectious complications, and treatment tolerance was measured.

**Results:**

After 7 patients completed the protocol, any differences were observed between groups in an interval analysis. Mucositis, pain, and tolerance was similar in both groups.

**Conclusions:**

Our results must be interpreted with caution due to the reduced sample size, but the use of bioadhesive chlorhexidine gel 0.2% didn’t contribute clinical improvement to the oral mucositis induced by radiation therapy and chemotherapy.

**Key words:** Chlorhexidine, mucositis, head and neck cancer.

## Introdution

Oral mucositis (OM) is considered one of the major debilitating side effects of cancer therapy due to the direct radiation of the mucosa with radiotherapy or by the effect of chemotherapy (1,2). This illness is described as the result of inflammation changes in the epithelial and sub-epithelial cells irradiated or affected by the treatment, causing discomfort in the patients because of the generalized erythema, frank ulceration, and denudation, associated with local pain as well, which constitutes the most important clinical manifestations of oropharyngeal mucositis (1,3). The induced pain makes it difficult for patients to eat, swallow, speak, or perform oral hygiene measures. These effects result in weight loss, dehydration, and a risk of oral infections (1).

Today, radiotherapy (RT) and chemotherapy (QT) constitute as one of the most commonly used combinations of therapies for head and neck cancer (H&NC). It has been demonstrated in previous studies that the association of these types of treatments result more effectively than using radiotherapy alone. Actually, the most frequent treatment is the programmed sessions of QRT, being necessary in most cases of surgical intervention too. The treatment must be uninterrupted; it has been demonstrated that the proliferation risk of residual tumor cells is incremented when the radiotherapy or the programmed chemotherapy is abandoned or interrupted, causing recurrences of the tumor and affecting the life quality of the patient (4).

Ninety percent of patients with head and neck cancer receiving standard radiotherapy and chemoradiotherapy will develop oropharyngeal mucositis (3), varying the incidence according to the oncology treatment schedule (5).

Intraoral lesions are commonly localized in non-queratinized oral mucosa, such as lip and buccal mucosa, lateral and anterior mucosa of the tongue, floor of the mouth, and soft palate. The hard palate and gums seem to be less susceptible to the effects of chemotherapy and radiotherapy (6).

In a conventional radiotherapy, the first radiation dose (10 Gy) causes a hiperqueratinized lesion, where a whitish appearance can go unnoticed. An early sign of OM includes erythema, which appears after approximately 20 Gy of cumulative radiation dosage for head and neck tumors. After 7–10 days, or a cumulative dosage of 30 Gy, ulcers covered by a pseudo membrane are detected; these pseudo membranes bring on the bacterial colonization with a high risk of sobreinfection, which are associated with discomfort and changes in patient dietary habits. Radio-induced OM lasts for at least 2 to 6 weeks after radiation therapy has finished (7,8).

OM induced by chemotherapy is usually more aggressive than that caused by radiotherapy. After 5-8 days of treatment, erythema appears, and 2 days after, edema and ulceration can be observed (7). Erythema is observed around the fifth–eighth day of treatment, and in the following days, edema and ulceration can be observed. After the end of the chemotherapy treatment, the mucosa will need about 7-10 days to recover completely (7).

Nowadays, there are a large number of treatments that we can choose from, but the strategies to reduce oral mucositis are still unclear. Due to the importance of the OM, many studies have been carried out. Different techniques have been described like intensive oral care protocol, anti microbial agents, anti inflammatory agents, citoprotectors agents, nutritional supplements, bioestimulants agents, or natural and homeopathic agents. Although all of these treatment options exist (6,9-11) to prevent and treat mucositis, there is no gold-standard protocol that is prominently better than the rest because there is not enough evidence describing a treatment with proven efficiency to surpass the other treatments for this condition (6,9,12-14).

Chlorhexidine is approved for use as an antibacterial mouthwash at a concentration of 0.12% and 0.2% to prevent the buildup of dental plaque and to prevent gingivitis (9,15). Its broad spectrum of antibacterial activity, minimal systemic absorption, and ability to bind to oral surfaces led to the use of prophylaxis in an attempt to prevent the development of oral mucositis (16). However, it has some disadvantages such as the discoloration of teeth, the bitter taste, and the unpleasant sensation (17,18).

The aim of this pilot study was to evaluate, in an initial way, the effectiveness of bioadhesive chlorhexidine gel 0.2% versus placebo as a preventive and therapeutic intervention of oral mucositis induced by radiation therapy and chemotherapy in patients diagnosed with head and neck cancer treated with chemoradiotherapy.

## Material and Methods

- Patient Characteristics and place of study

The pilot study was conducted in the oncology department of the University Hospital “Virgen del Rocío” in Seville after approval from the local Internal Review Board. This was a prospective, placebo-controlled, randomized, and double-blind pilot study.

The inclusion criteria was the following: patients aged 18-65, who had histological documented diagnosis of squamous carcinoma on the head and neck region in stage III and IV according to the TNM classification and who received combined radiation treatment (conventional fractionation 70 Gy reaching the tumor and affected lymph nodes, and 50 Gy at the nodal areas drainage in 9 weeks) and chemotherapy (cisplatin 100 mg/m2 IV on days 1, 22, and 43 of irradiation). The patients voluntarily expressed their intention to participate in the clinical trial with informed consent before enrolling in the study.

The exclusion criteria was the following: patients who have hypersensitivity or an allergy to any of the components included in the study, patients with HIV, diabetes and autoimmune diseases, and those who do not fulfill all of the inclusion criteria mentioned above.

After carrying out this pilot study, 7 registered patients, 4 in the study group, and 3 in the control group, the study was stopped. All the patients fulfilled the pilot study.

- Study groups 

Experimental/study group: In the protocol of treatment, these patients performed topical application with bioadhesive chlorhexidine gel 0.2%. In the control group, these patients used a placebo gel in the way that we specify below.

- Study design

The study protocol consisted of the administration of a multivitamin that contained vitamin E and Zinc Sulfate, the performance of rinses with 15 ml of Benzydamine for 2 minutes, 5 times a day, and the local application of ice on the oral mucosa at least for 20 minutes on days 1, 22, and 43 of cancer treatment (after chemotherapy with cisplatin), besides the application of radiotherapy for 9 weeks (exposed previously).

Simultaneously, topical application was performed with bioadhesive chlorhexidine gel 0.2% in the study group and placebo gel for the control group in 5 applications per day from the time of initiation of cancer treatment to 2 weeks after completion of chemo-radiotherapy treatment (total time of study was 77 days, 11 weeks). The patient was instructed in the placement of the gel (placebo or chlorhexidine) with a syringe by extracting a dose of 10 ml for each application, 5 times per day, after rinsing with benzydamine.

The randomization and double-blind trial was carried out as follows. Both gels were prepared by LACER laboratories in Barcelona (Spain). The needed hardware was added for all samples to have identical color, flavor, and texture, which in turn would be comfortable for the patient. Patients were randomly assigned to receive either protocol of oral care with chlorhexidine or placebo by selection of a sealed envelope (randomized list generated by computer).

The patient scored daily in a booklet provided below for the onset of pain and its intensity before applying the gel, immediately after, and 3 hours later (using a visual analog scale numbered from 0 to 100) in the first application of the gel during the day. It was also noted in this booklet if the use of analgesics and/or anti-inflammatory oral mucositis was required, and in what quantity (number of pills and doses).

The study was planned and carried out in compliance with the Declaration of Helsinki and Good Clinical Practice. The pilot study protocol was approved by the Experimentation Ethics Committee (EC) of the University of Seville and financed by the Andalusian Health Service (Code SIGI- S0095).

- Clinical assessment protocol

Gradation, occurrence, and remission of mucositis

The integrity of the mucosa was assessed using the WHO criteria for the grading of oral mucositis on a scale of 0-4, where 0 is “no change,” 1 corresponds to “erythema and/or pain,” 2 “ulceration and ability to eat,” 3 “ulceration and limited ability to eat,” and 4 “ulceration with hemorrhage and necrosis.” The examiner is always the same to avoid the variability between different examiners’ criteria.

Initial exploration of the oral mucosa is recorded before the beginning of chemo-radiotherapy, and subsequent reviews were carried out weekly until 2 weeks after the full cancer treatment of the patients.

- Pain

Patients rated pain sensation following a visual analogue scale (VAS) flat with values ranging from 0 (“no pain”) and the value 100 (“unbearable pain”). This annotation was made at home once a day throughout the study, before applying the gel, immediately after, and 3 hours after (first daily gel application).

- Adjuvant analgesics

Patients recorded in the same booklet if they took analgesics or anti-inflammatories due to mucositis, and they specified the amount of drugs required on a daily basis.

- Treatment tolerance

The evaluation of tolerance was based on the frequency with which patients have had at least one adverse event during the trial. The patient and investigator will score the global tolerance at the end of treatment by an oral 5-point scale: 5 = very good, 4 = good, 3 = moderate, 2 = poor, and 1 = very poor.

- Infectious complications

The investigator assessed the occurrence of complications due to infection of the lesions of mucositis in weekly reviews during treatment.

- Data analysis

Data were analyzed using SPSS 9 for Windows Support Unit Research Hospital Universitario “Virgen del Rocio.” We used the chi-square test for the analysis of the data obtained in the comparison of qualitative variables and Mann–Whitney U test for comparison of means.

## Results

In this pilot study, 7 patients with head and neck carcinoma treated with chemo-radiotherapy were randomized and included in the intention-to-treat population for efficacy analyses. The patients consisted of 5 men and 2 women with mean age of 59.57 ± 10.97 years. In the control group, 3 patients were treated with a mean age of 63.3 ± 15.89 years. In the experimental group, 4 patients were treated with an average age of 56.75 ± 6.90 years ( Table 1). All patients were undergoing treatment chemo-radiotherapy.

**Table 1:**
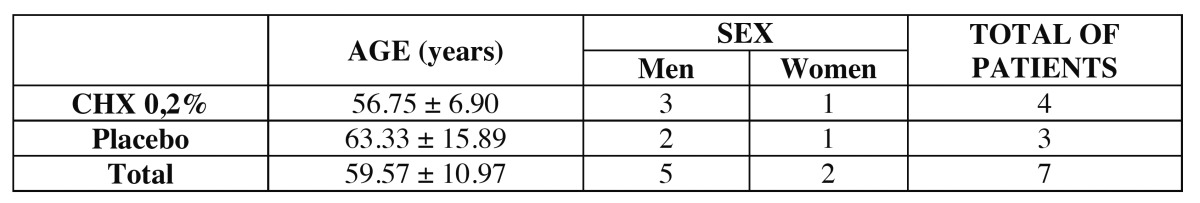
Characteristics of the patients included in the study.

Regarding the frequency of the hygienic habits, 4 patients had a frequency of 3 times per day, 2 patients 2 times per day, and 1 of them 1 time per day. However, in terms of harmful habits, only 2 patients had no such habit, 2 of them were ex-smokers, 2 were smokers and drinkers, and one of them was a drinker ( Table 2).

**Table 2:**
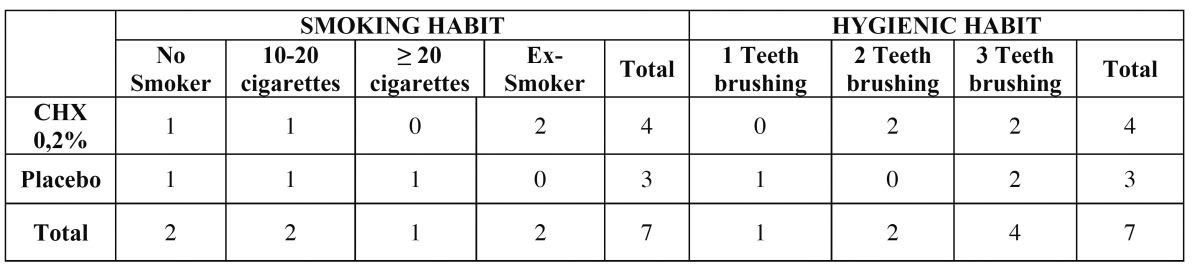
Habits of the patients included in the study.

The integrity of the mucosa to the WHO scale for mucositis was slightly higher in the study group than in the control group, but without obtaining statistically significant differences (*p*>0.05) ( Table 3).

**Table 3:**
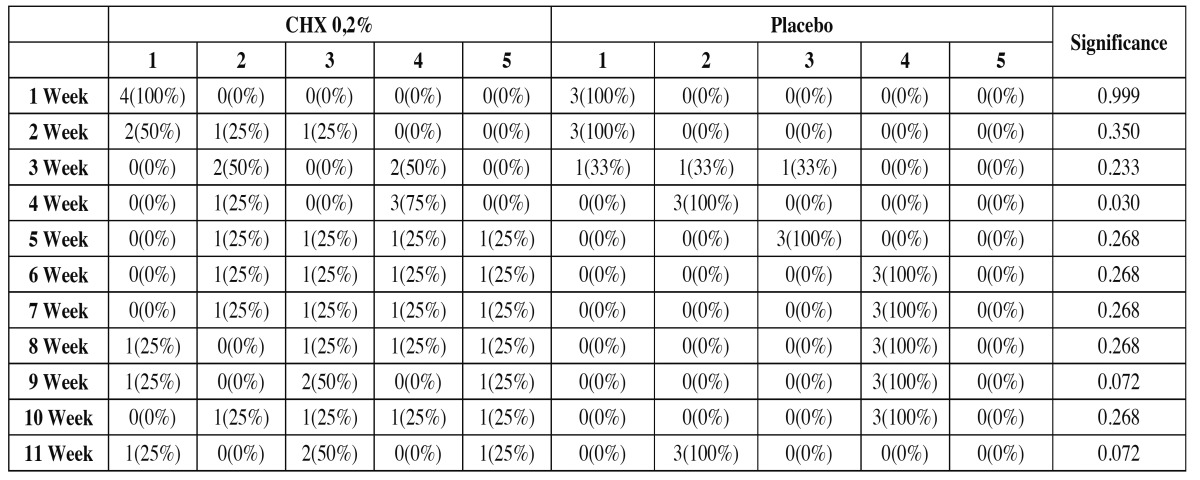
The integrity of the mucosa to the WHO scale for Mucositis along the study.

**Figure 1:**
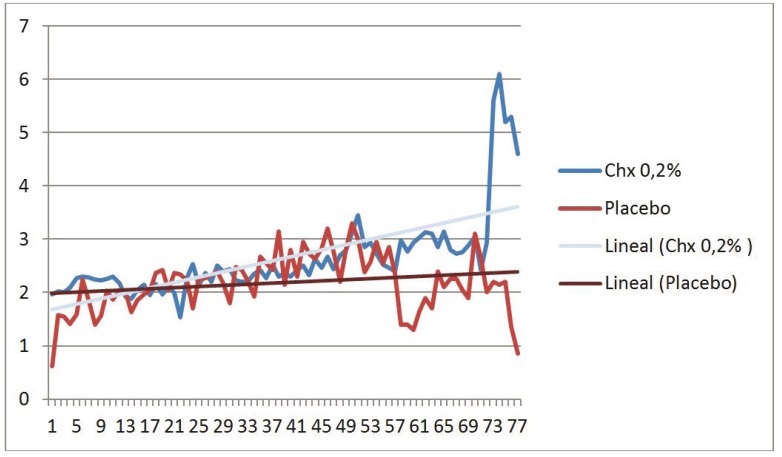
Pain prior to placement the gel in the study group than in the control group.

**Figure 2:**
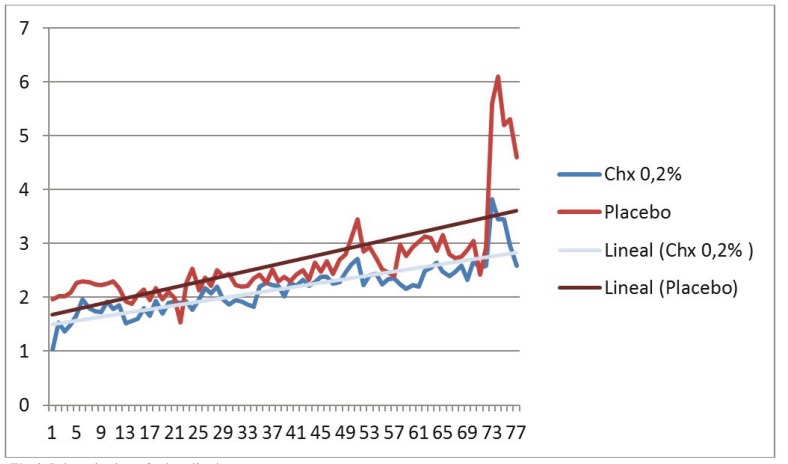
Pain at the time of gel application.

**Figure 3:**
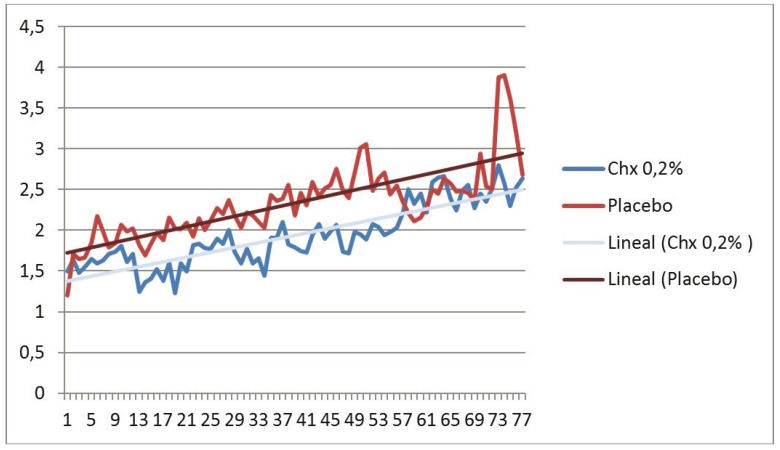
Pain one hour after the application.

In relation to the pain associated with the application of gel, a greater degree of pain prior to placement in the study group than in the control group was observed (Fig. 1), although the pain at the time of gel application and 1 hour later of placement was less in the study group versus the control group (Figs. 2,3). However, no differences in trends were found between the chlorhexidine and placebo.

The tolerance of the treatment from the patients’ and researchers’ point of view had the same punctuation on the scale from 1 to 5. In the study group, the tolerance was slightly better than in the control group ( Table 4). Moreover, infectious complications were similar in the experimental group than in the control group ( Table 5). No statistically significant differences were found in any of the two variables (*p*>0.05).

**Table 4:**
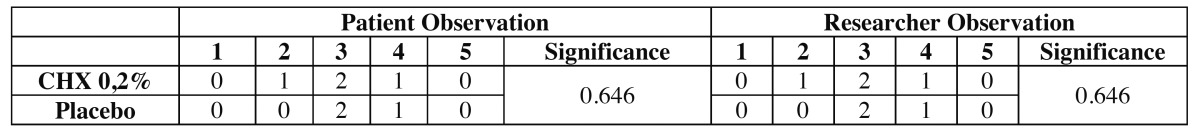
Tolerance of the treatment from the Patient and the Researcher View.

**Table 5:**
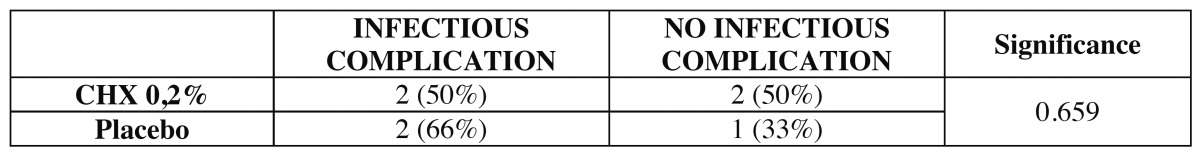
Infectious Complication on the study and control group.

## Discussion

This is a report of a pilot study. The calculations of the sample that were necessary to extrapolate the data once they were drawn from the study sample were carried out and the total number of patients was calculated as 96. However, the authors have decided not to advance in this final study. The reasons for this included the very limited effectiveness (if some are present) of the drug used in the study group (chlorhexidine gel 0.2%) compared to placebo.

Data from several randomized clinical trials show that chlorhexidine rinses do not have a major impact on the prevention of mucositis in patients undergoing radiotherapy (18,19). However, it significantly seems to reduce oral inflammation and ulceration in patients undergoing chemotherapy. Chlorhexidine rinses are treatments that are applied across the surface of the oral cavity without acting specifically on lesions, so the use of chlorhexidine gel would seem to have a more advantageous indication for the treatment of injuries caused by mucositis because of the localized application (20).

In opposition to the initial hypothesis, our results don’t show a clinical improvement of the study group’s patients. The use of bioadhesive chlorhexidine gel 0.2% does not reduce the frequency of mucositis in the oral cavity or the pain caused by mucositis induced by cancer treatment, and with no significant differences between the patients’ pain before and after the application of it. These results are in agreement with other work with chlorhexidine rinse, as in the paper presented by Rutkauskas and David (21), who investigated the effect of chlorhexidine versus a placebo in patients undergoing haematopoietic stem cell transplant (HSCT) or remission-indication chemotherapy. The study showed chlorhexidine to be ineffective in preventing mucositis. Also, Raether and colleagues did not support the use of chlorhexidine mouthwash for the prevention of mucositis in patients with bone marrow transplantation, and they found that there was no significant difference in the severity of oral ulceration between the chlorhexidine and placebo groups (22-24). However, there are studies that support prophylactic use of 0.12% chlorhexidine gluconate to reduce the frequency of oral mucositis and oral pathogens in children with Acute Lymphoblastic Leukemia undergoing antineoplastic chemotherapy (25,26). The preventive oral protocol using chlorhexidine mouthwash can reduce both the incidence and the severity of oral lesions in children suffering from leukemia receiving chemotherapy, according to these studies (25).

Nevertheless, it can be suggested from the data presented here that chlorhexidine may play a part in reducing oral damage during radio-chemotherapy, possibly through plaque control and a reduction in the oral microflora (26,27). There was no difference in mucositis between the groups although bacterial and fungal infections were found slightly less often among the patients using chlorhexidine. However, it cannot be translated into a reduction in the pain produced by mucositis. These results are in accordance with those published with chlorhexidine mouthwash (21).

Nowadays, numerous studies can be found about the treatment or the prevention of the mucositis with different products. Chlorhexidine mouthwash is a common product tested in the treatment of this illness, and not only in comparison with the placebo. Dodd *et al*. published some studies in which chlorhexidine was compared with sterile water (28) and another study in which a solution of Lidocaine, Benadryl, and Maalox (29) was the object of comparison. No differences in the severity of mucositis were found between the groups in those studies. However, there was a decrease in the number of microorganisms in the CHX groups compared with the control groups (30).

Chlorhexidine has been also compared with benzydamine hydrochloride oral rinses for the prevention and treatment of irradiation mucositis in patients with head and neck cancer (3). Significant differences were not detected between groups on outcome measures; a trend has emerged toward a lessening of oropharyngeal mucositis for patients who received benzydamine compared to patients who received chlorhexidine (3). However, these results contrast with those published by Cheng, who stated that from the patients’ perspective, chlorhexidine is more helpful than benzydamine in reducing mucositis and palliating oral discomfort (31).

Other products have been tested for the prevention and treatment of oral mucositis like zinc-containing mouthwash (25) or amine-stannous fluoride solution (32). None of these rinses have shown clear advantages over chlorhexidine so there is still no treatment for this complication of the treatment of head and neck cancer.

Currently, there is no effective therapy to resolve or substantially improve the symptoms of mucositis already established (9), although recent studies show that the use of a low energy laser can be a promising therapy (33,34).

It should be pointed out that the results of the present pilot study should be interpreted with caution due to the reduced sample size. Nevertheless, it seems that in agreement with the evidence indicating that chlorhexidine rinse is not useful for the prevention and treatment of the mucositis induced by chemoradiotherapy of head and neck cancer (27,30), the application of gel bioadhesive of chlorhexidine with the same intention has the same usefulness.

In our opinion, further studies will be required to find a treatment different from the chlorhexidine (rinse or gel) for mucositis and improve the quality of life of patients undergoing chemoradiotherapy.

## Conclusions

In this double-blind, randomized pilot study, the gel bioadhesive of chlorhexidine 0.2% has not provided better clinical results than a placebo on pain and discomfort in the oral mucositis caused by chemoradiotherapy in the treatment of head and neck cancer.
